# Temperature mediated habitat selection in sympatric deer species with varying body size: thermal cover and forage availability as potential drivers

**DOI:** 10.1186/s40462-025-00581-2

**Published:** 2025-07-22

**Authors:** Anna Widén, Joris P. G. M. Cromsigt, Annika M. Felton, Fredrik Widemo, Lukas Graf, Göran Ericsson, Navinder J. Singh

**Affiliations:** 1 Department of Wildlife, Fish and Environmental Studies, Swedish University of Agricultural Sciences, 901 83 Umeå, Sweden; 2 Department of Zoology, Centre for African Conservation Ecology, Nelson Mandela University, PO Box 77000, Gqeberha, 6031 South Africa; 3 Southern Swedish Forest Research Center, Swedish University of Agricultural Sciences, Box 190, 234 22 Lomma, Sweden

## Abstract

Knowledge about habitat selection is crucial for ungulate management and conservation to handle competing land use. Thermal tolerance and access to food are two important drivers of habitat selection. Thus, moving into thermal shelter when temperatures increase may result in reduced energy intake e.g. mature forests providing thermal shelters through high canopy cover, but lower amounts of forage than more open habitats. Body size affects heat sensitivity, with larger animals being more sensitive. In this study, we investigated ambient temperature mediated habitat selection in three differently-sized, sympatric, deer species (moose, red deer and roe deer) during spring and summer in Sweden. We also assessed the trade-off between thermal shelter and forage availability. We used data from GPS-collared animals with a combination of land cover and airborne LIDAR data to quantify canopy cover as a proxy for thermal refuge and shrub cover as a proxy for forage availability. We found that temperature influenced habitat selection in all three deer species however with divergent patterns. During warmer daily temperatures, moose and red deer selected areas with more canopy cover for thermal shelter, however showing opposite patterns during warmer nights, suggesting patterns of thermoregulatory behaviour. Roe deer showed no influence of high temperatures on canopy cover selection however,, selected more strongly for areas with high canopy cover during colder temperatures i.e. showing contrasting patterns compared to moose and red deer, suggesting that canopy cover may be relatively less important for roe deer as temperature increase. All three species selected for shrub cover during warmer temperatures, which can provide both high forage availability and shade over bed sites for smaller deer species. Our findings indicate that canopy cover and shrub cover habitats appear to be important as temperatures increase, but their significance varies among species. Furthermore, our study highlights the complexity behind habitat selection in sympatric ungulate species, food intake and predation risk.

**Supplementary Information:**

The online version contains supplementary material available at 10.1186/s40462-025-00581-2.

## Introduction

Habitat selection by animals determines their distribution across the landscape and, thus, how they affect ecosystems and human land use [1, 2]. Human land use may be negatively affected by free-ranging large herbivores, such as ungulates, via grazing on farmlands or browsing in managed forests [3, 6]. At the same time, ungulates play an important role in the ecosystem by maintaining heterogeneity and supporting the diversity of flora and fauna [7, 8]. Knowledge of the drivers behind ungulate habitat selection is therefore crucial for land use management including ecosystem restoration and conservation [7, 9–12].

Habitat selection is governed by different factors in a hierarchical manner, where forage availability is one key factor influencing habitat selection on several scales [13, 16]. Apart from finding food, animals also select for thermal cover to avoid overheating [17, 18] and select certain habitats to minimize predation risk [19]. The influence of temperature on the behavior of cold-blooded species (ectotherms) has been studied in captivity for a long time [20, 22]. Direct effects of temperature on the spatial behavior in free-ranging warm-blooded species (such as ungulates) are also well studied [18, 23–27, 95].

Temperature can both influence animals´ daily activity patterns, e.g., by forcing them to be more active during the night when it is cooler [28, 30], and influence their habitat selection, by choosing habitats that provide more suitable microclimates/temperatures [18, 25, 31]. This can include seeking cooler habitats that provide thermal shelter when ambient temperature is high [17, 32–35].

Behavioral adjustments in habitat selection as a function of temperature have been observed in several different ungulate species [36]. For example, in alpine environments, alpine ibex (*Capra ibex*) use the altitudinal gradient as a way of escaping warm temperatures during summer and move to higher elevation when temperature increases [28, 37]. Elk (*Cervus elaphus roosevelti*) and black-tailed deer (*Odocoileus hemionus*), living in forested landscapes, select mature old growth forests during warm days [25], Merrill 1991). Similar patterns have been observed in moose (*Alces alces*), selecting dense canopy forests when temperature increases [17, 18, 35].

Changes in habitat selection, as a result of temperature, may come at a cost of losing foraging opportunities [18, 38]. Habitats providing more suitable microclimates during higher temperatures, such as forests with dense canopy cover, may provide less forage than habitats with light canopy cover [39]. Thus, during times of unfavourable temperatures, animals may trade-off food availability and quality for thermal cover [17, 18, 39]. However, in some ecosystems habitats providing thermal refuge may also provide high quality forage, for example in cases of moose bed site selection in riparian areas that provide high quality forage [40].

Moreover, since temperature tolerance is influenced by body size [32, 41, 42], the influence of temperature on spatial behavior and habitat selection may differ among species. Larger species have a lower body surface to body mass ratio, and can more easily retain heat when it is cold, but may have difficulty losing heat when it is warm [42]. Because of this difference in surface-volume ratios, larger ungulates are more prone to overheating, limiting their activity more than small ungulates at warm temperatures, whereas smaller ungulates are more susceptible to cold stress [43, 45]. Additionally, large mammals often use physiological mechanisms to cope with heat stress, such as sweating or panting [46]. However, these coping capabilities may differ between deer species, i.e. moose lack the ability to sweat and thus rely on panting or thermoregulatory behaviour [17, 47], in contrast to red deer that possess extensive sweating capabilities [48].

Due to these differences in thermal tolerance among species, the trade-offs between selecting habitats for food versus thermal cover may also vary among species. To increase our understanding of this, we studied these trade-offs for three of Europe’s most common, sympatric, wild ungulate species, moose, red deer (*Cervus elaphus*) and roe deer (*Capreolus capreolus*). These species span a 15-fold size range from 20 kgs for an average female roe deer to about 300 kgs for a female moose.

The differences in the distribution of our three study species may be at least partly due to these three species having a different thermal tolerance, with moose only being distributed in northern Europe, while red deer and roe deer are distributed across the entire central and southern Europe [49, 50]. Moose, the largest extant wild ungulate in northern Europe, is known to be especially heat sensitive [36, 51–53]. Their thermal tolerance appears to vary somewhat between studies, with different temperature thresholds being reported for onset of heat stress. [54] reported that moose thermal limits start at around 4 degrees Celsius in summer, and −5 degrees in winter, and open mouth panting starts at 20 degrees in summer and 0 degrees in winter. However, more recent studies have identified a range of temperatures at which moose begin to show thermoregulatory behavior, suggesting free-ranging moose do not have static limits of ambient temperature at which they become heat stressed [24, 51, 55, 56]. Thermal limits have not been determined for roe deer, but are suggested to be above 25 °C in central Europe [57], higher than the upper critical limit for moose. For red deer, respiratory rate as a function of temperature seems to increase somewhere between 20 and 30 °C [48].

Moose, red and roe deer also differ in dietary preferences which may influence their habitat selection. While moose and roe deer are classified as browsers, while red deer are categorized as mixed feeders [58, 59] with a relatively larger part of their diet composed of grass [59–61]. However, the diet of red deer overlaps to a large extent with diets of roe deer and moose in our study area, as the diet of all three species include large proportions of dwarf shrubs and deciduous tree species [59]. Until now, studies investigating temperature mediated habitat selection of northern hemisphere ungulates have focused on single species separately (e.g. [17, 18, 24, 25, 40]). However, studies on sympatric species have been conducted on southern African ungulates (e.g., eland (*Tragelaphus oryx)*, impala (*Aepyceros melampus*), arabian oryx (*Orys leucoryx*) and blue wildebeest (*Connochaetes taurinus)*), (such as [62, 64]). Increased knowledge of habitat selection and landscape use in a multispecies system is important in order to gain information about how drivers behind habitat selection may influence species interactions and their population dynamics. Furthermore, species co-occurring in the same area may compete over space and resources, and therefore influence their individual and combined impact on the landscape [5, 65]. Quantifying thermoregulatory behavior is also a necessary step in order to evaluate global warming effects on the distribution and dynamics of species communities [66, 67].

In this study, we addressed how temperature influences habitat selection in moose, red deer and roe deer, in an area dominated by human land use (agriculture and forestry) in northern Sweden. We linked GPS positions from marked individuals of all three species to environmental covariates regarding land use types (land cover data) and canopy cover (airborne laser scanning data, ALS). We also incorporated the possible influence of time of day (daytime versus night-time) in our analysis, since the potential influence of temperature on habitat selection may vary depending on the time of day [18]. Furthermore, we investigated the potential trade-off between forage and thermal cover by incorporating data on forage availability by using ALS data.

We predicted that temperature has a direct influence on selection, by making animals select habitats that provide cover when temperature increases. This effect should be strongest for the largest species, moose. Additionally, we expect to find support for a food-cover trade-off. Hence, during colder temperatures, animals should select for habitats providing high forage availability. On the contrary, when temperature increases, we expect stronger selection for habitats providing canopy cover rather than maximizing forage availability. Again, we expected this trade-off to be strongest for the moose as the largest species of the three.

## Methods

### Study area

**Fig. 1:**
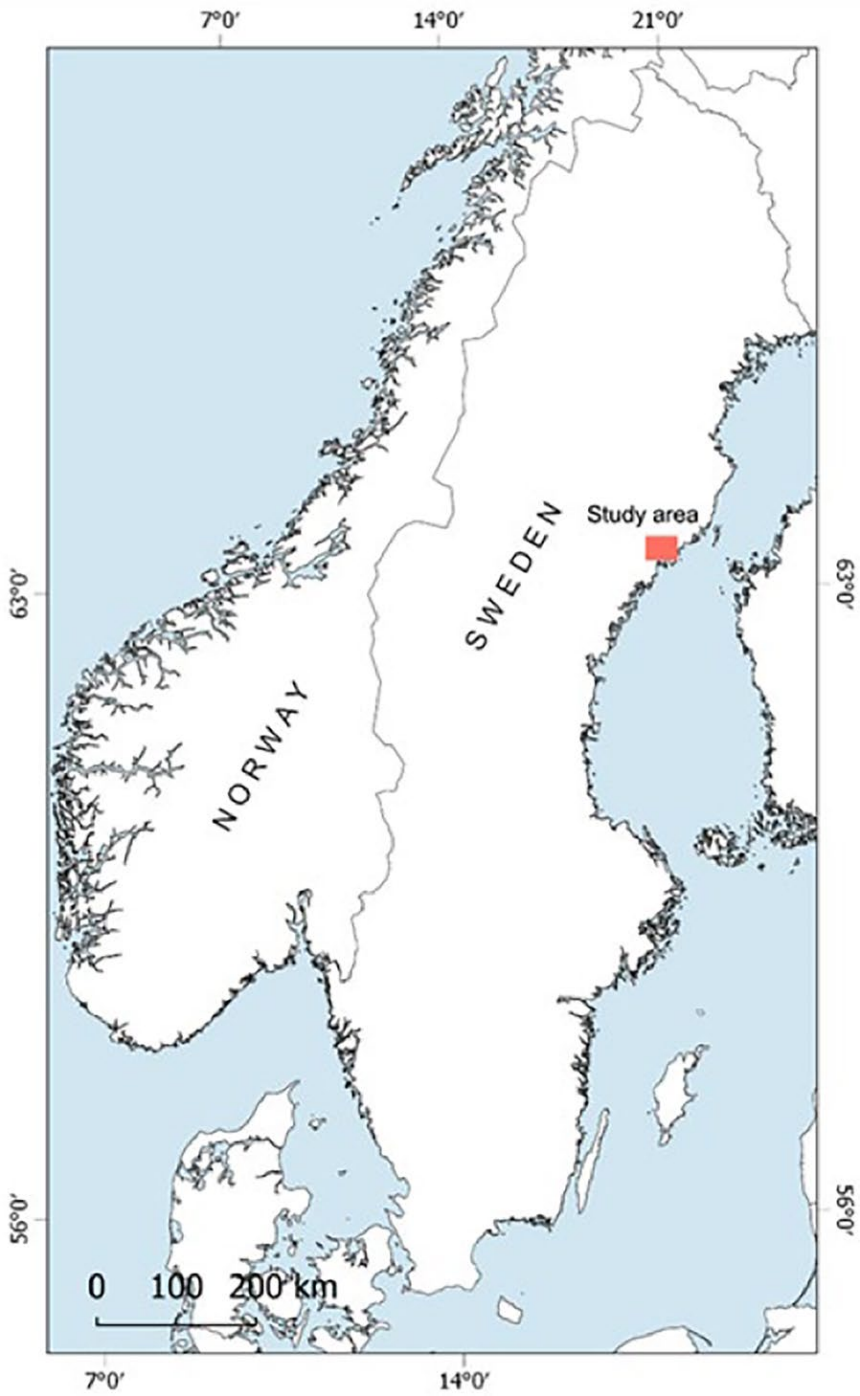
The placement of the study area where positions of GPS collared animals were studied

The study area is located in northern Sweden in Västerbotten county (lat: 63.4351, lon: 19.6731, Fig. 1). Västerbotten county is dominated by forest land (including mires and wetlands), comprising up to 70% or the land surface, where arable land only comprises 1% of the land area [68]. Common tree species include Scots pine (*Pinus sylvestris*), Norway spruce (*Picea abies*), birches (*Betula spp*.), poplars (*Populus spp.),* and willows (*Salix spp*.). Ericaceous shrubs, such as of the genera *Vaccinium*, *Calluna*, and *Empetrum*, together with mosses and lichens dominate the field layer. Of the arable land, the majority (~ 90%) is grass, ley and fallow and only 10% is cereal crops of different kinds [69].

Moose, roe deer, red deer and fallow deer (*Dama dama*) occur sympatrically in the area. Semi-domesticated reindeer (*Rangifer tarandus*) occur during the winter. Predators occurring in the area are brown bear (*Ursus arctos*), lynx (*Lynx lynx*) and red fox (*Vulpes vulpes*), all potentially predating on either adult ungulates or their offspring. There has also been occasional visits by wolverine (*Gulo gulo*) and wolf (*Canis lupus*) in the study area.

The mean monthly temperature during the study period (May–September 2017–2019) ranged between 6.3 °C for the coldest month and 19.6 °C for the warmest month, with a maximum temperature of 28.8 °C and a minimum temperature of −2.7 °C. The monthly precipitation ranged between 8 and 76.8 mm [70].

### GPS data

In total, 48 animals, (27 moose – all females, 7 red deer – all females and 12 roe deer (all females) were captured and equipped with GPS collars (Vectronic-Aerospace, GmbH, Berlin, Germany). For moose we followed our standardized protocol for immobilization procedures using a CO_2_ powered rifle (DANiNJECT, Kolding, Denmark) from helicopter [71, 73]. Red deer captures followed similar protocol, but were sedated through ground darting at bait sites with a drug combination of tiletamin-zolazepam 200-250 mg (Zoletil® 100 Vet., Virbac, Carros, France) and xylazine 200–250 mg (Xylased® 500 mg, Bioveta, Ivanovice na Hané, Czech Republic) [74]. Roe deer were caught in approved wooden box traps (L6 Rådjursfälla M/Öster Malma) baited with forage for deer and were handled unsedated (see [75]). Moose and roe deer were monitored by collars during 2017–2019, while red deer were monitored in 2018 and 2019. GPS locations were recorded at intervals varying between 30 min and 3 h for moose, while 1 to 3 h for roe and red deer. In addition, all GPS collars were equipped with a temperature sensor measuring ambient temperature (collar temperature, Tc). All temperature sensors were calibrated by the manufacturer and checked so that they corresponded to ambient temperature before use. All GPS locations were stored and quality controlled in the WRAM database (Wireless Remote Animal Monitoring plat-form) [76]. We used data from 1 May – 30 September to account for the period with maximum forage availability and to capture the growing season. The dates were selected based on information about the start and end of the vegetation period in our study area [77]. Hence, during this period, forage is not a limiting factor but is abundant in the entire area.

Within our study period, moose is hunted from 1 September using hunting dogs. Red deer and roe deer are hunted from 16th of August. However, it is only allowed to hunt female red deer and calves, or roe deer males. Stalking is the only allowed method [78].

### Ethical consideration

The project was approved by the Ethics Committee Animal Experiments for Northern Sweden in Umeå Dnr: A14-15, A3-16, A28-17, A44-16, A11-20 and all captures were conducted following Swedish laws concerning animal research ethics. Furthermore, all personnel were trained and certified according to the standards of the Swedish Board of Agriculture.

### Environmental covariates

To investigate habitat selection by moose, red deer and roe deer in response to Tc we used a combination of data sets that we expected to influence habitat selection in relation to temperature in large ungulates [55]. We used National land cover data from the Swedish Environmental Protection Agency [79], to get information about land cover types, which is mapped at a spatial resolution of 10 m. The land cover data contains 26 classes derived from a combination of satellite and laser scanning data. The land cover classes have been ground truthed by comparing the categories with field inventory data from the National Inventories of Landscapes in Sweden (NILS) and the Swedish National Forest Inventory with results showing a 94–95% overlap in categorization [80]. We reclassified this raster into 4 new land cover categories: Early successional forest, arable land, forest and “other”. Early successional forest was defined using the NMD classification and is open non-vegetated land as well as regenerating forest where trees are below 5 m. Forest was defined as any type of forest land where trees are above 5 m. “Other” was defined as all other open land such as urban, water and wetland (see appendix for full classification). Waterbodies were included in the other category due to constraints on the number of categories we could analyze within our sample size limitations. Additionally, only a small percentage of GPS positions fell within water bodies for our three species, 0.75% for moose, 2% for red deer and 1.7% for roe deer. These different land cover types have different qualities as thermal refuge or providing forage, but also as important hiding cover from predators (Table 1).

**Table 1:** Properties/qualities of thermal refuge, hiding cover/predator concealment and forage availability for each land cover type during the study period (i.e. the growing season).

	Thermal refuge	Hiding cover/predator concealment	Forage availability	Explanation
Forest	Day: High Night: Low	Low-medium	Medium	Mature forests with trees > 5 m. Diverse horizontal vegetation layers provide hiding cover and forage. Some areas offer high forage availability; others less
Arable land	Day: Low Night: High	Low	High	Open habitat comprised of crops or grass. High quality forage during growing season. Low canopy cover and shrub cover
Early s. forest	Low-medium	High	High	Young forests or clear cuts with trees < 5 m. High forage availability and cover serving as predator concealment
Other	Low	Low	Low	Urban areas and water create a diverse category. However, since urban areas dominate, refuge and forage availability is generally low

The qualities of each land cover type are defined as low–high based on our knowledge of the study system where high implies that the land cover type generally provides a lot of thermal refuge (here meaning refuge from high temperatures) or hiding cover for example. Note that for some land cover types the quality of thermal refuge can differ, depending on time of day. For each land cover type there is a more detailed explanation about the different properties of that habitat and potential complexity of diverse habitat groups. To get information about these land cover types we used National land cover data from the Swedish Environmental Protection Agency [79], Early s. forest stands for early successional forest

We used data from Airborne Laser Scanning (ALS) from the Swedish National Land Survey to calculate canopy cover [81]. Canopy cover was calculated for the entire study area, hence, in all land cover types. The ALS data from the study area was collected in 2020. We used data from 1156 scanning squares of 2.5 × 2.5km2 in six different scanning blocks. Blocks were scanned using an ALS80-HP scanner from Leica. ALS data was scanned at 3000 to 3200 m, minimum point densities ranged between 1.1 and 1.23 points/m^2^. Overlap between flight lines was between 10 to 11%. We processed ALS data using the lidR – package [47]. We only used first returns [82, 83] to calculate variables from the ALS data. First, we calculated canopy cover as.

**Fig. 2:**
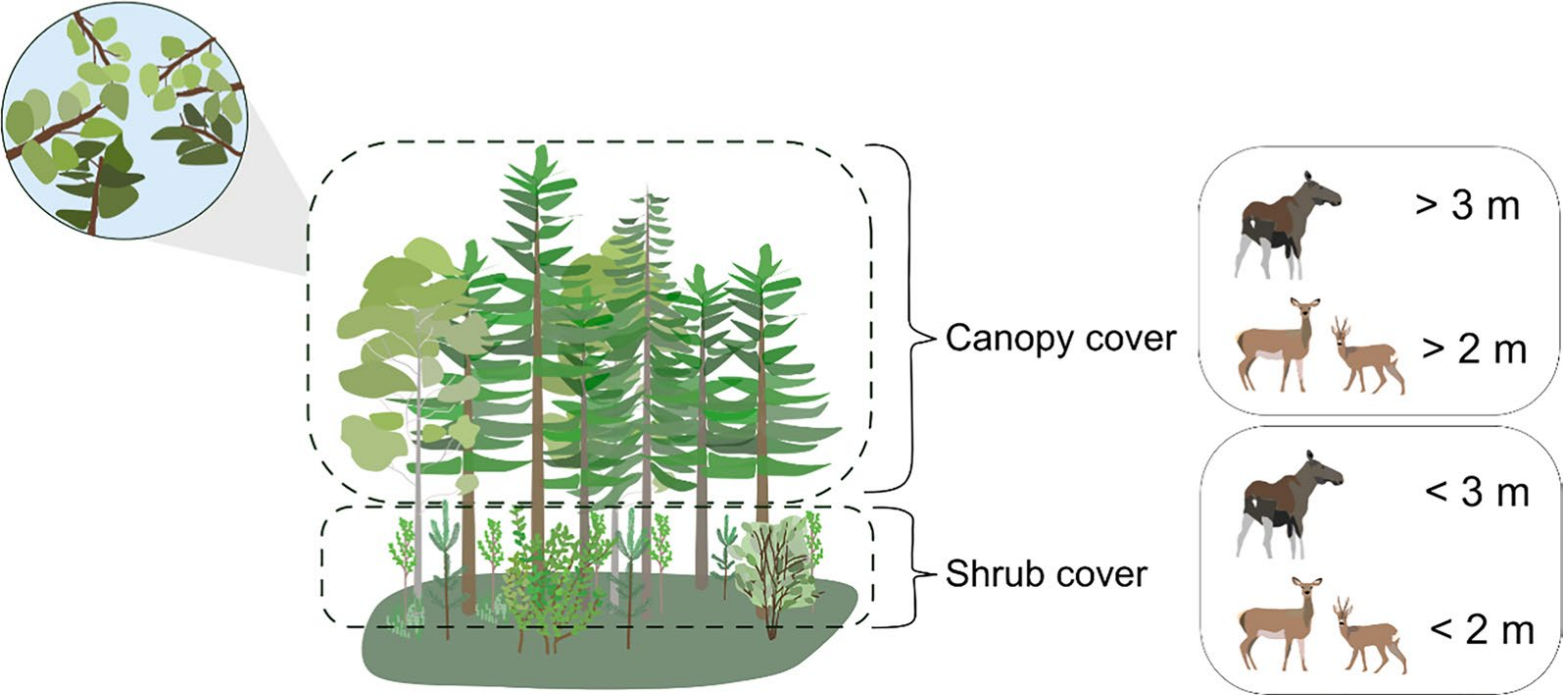
Visual representation of canopy cover and shrub cover classification for the different species. We used a 3 m height threshold to calculate canopy cover for moose, whereas we used a 2 m height threshold for both roe and red deer, which we expected to reflect their maximal browsing heights. Canopy cover was calculated above the respective height threshold, whereas shrub cover was calculated below the respective height thresholds

$\frac{first \,returns \,above \,x \,meters}{all \,first \,returns}$, where x is the number of first returns above 3 m (for moose) and 2 m (for red deer and roe deer) (Fig. 2). This generated pixel values ranging between 0 and 100% being the percentage (%) of echoes above 3 and/or 2 m. E.g. a canopy of 100% means that 100% of the echoes were above 3 and/or 2 m, i.e. no echoes from lower strata, while a canopy cover of 0% means that none of the echoes were above 2 or 3 m, hence, being open land or an area with lower trees. We chose 3 and 2 m respectively to represent canopy cover that was above each species browsing height, i.e. not serving as food and being high enough to be above each species head while standing up (Fig. 2).

We further calculated shrub cover from the ALS data, which we considered as a proxy for forage availability for ungulates using the formula.

$$\left(\frac{\% all first returns below x m}{\% all first returns below 0.5 m}{/}_{\% all first returns below x m}\right)$$, where x is the % of first returns below 3 m (for moose) and 2 m (for red deer and roe deer), corresponding to their browsing heights (Fig. 2) [59, 84]. This generated pixel values ranging between 0–100% being the percentage (%) of echoes below 3 m and 2 m respectively. Similar to canopy cover, shrub cover was calculated in the entire study area, hence, in all land cover types.

To match the spatial resolution of the National land cover data, we spatialized both canopy cover and shrub cover with a 10 × 10 m spatial resolution. We added a 20 m buffer around each scanning square to avoid edge related artifacts in our wall-to-wall output raster [47].

### Data analysis

#### Ambient temperature

We used collar temperature (Tc) as a proxy for ambient temperature in our habitat selection analyses. The Tc captures the spatial and temporal variation in temperature on a much more detailed scale than weather station data. Furthermore, previous studies have used Tc as an index of ambient temperature when investigating habitat selection [18] based on a strong correlation between Tc and weather stations data, where they found an average correlation of 0.91 between collar and ambient temperature of the nearest weather station [85]. We used Tc as a continuous variable since earlier research has suggested that moose show response to heat stress at different temperatures [24, 51, 86], and that free-ranging moose seem to not have static limits of temperature at which they become heat stressed [55, 56].

#### Habitat selection

We analysed habitat selection using integrated Step Selection Functions (iSSF) [87] to analyse whether selection for land cover, shrub cover and canopy cover is dependent on temperature. Using iSSFs enabled us to analyse habitat selection at the end of the step while accounting for movement related parameters. Before fitting the iSSFs the GPS data was cleaned to a threshold of minimum 4 satellites to ensure reliable location accuracy and reduce the influence of positional error on movement metrics. We homogenized sampling rates by resampling to a three-hour sampling rate for roe deer and one-hour for moose and red deer. We fitted iSSFs for every individual using the amt-package [88], generating 50 random available steps [89] for every observed step. Available steps were generated using a gamma distribution to fit step length and a Von Mises distribution for turning angles. We extracted environmental covariates at the end of each step and matched it with the Tc at the start of the step in order to evaluate how animals select for habitat as a response to temperature. In other words, we evaluated how habitat selection at the end of the step was influenced by temperature at the beginning of the step. We used canopy and shrub cover as continuous variables in the model in addition to the land cover class (with the four categories: arable, early successional forest, forest and other). We used forest as the reference habitat class. We screened variables for correlations using *pearson’s* correlation coefficient r (Table 9 Appendix).

We performed z-score transformation on canopy cover, shrub cover and ambient temperature [90]. We further included the step length, the logarithm of the step length and the cosine of the turning angle as covariates in the model to account for the underlying movement process of iSSFs [87].

To assess whether circadian rhythms potentially influence habitat selection, we included information about day and night according to light conditions (day = from sunrise to sunset, night = from sunset to sunrise). Each GPS location was categorized as day or night using data on light conditions from the US Naval Observatory (https://aa.usno.navy.mil/).

We analysed individual iSSFs using conditional logistic regression [91] to obtain parameter estimates of habitat selection at the individual level, using the matched sets of observed and available steps as the response variable. To avoid convergence issues, we ran separate models for day and night and species. The final model included canopy cover, shrub cover, land cover type, and one-way interaction between Tc (collar temperature at the start of each step) and land cover, canopy cover and shrub cover. For some individuals, the interaction between land cover type and Tc led to convergence issues, in which case we removed the land cover type from the model (n = 19). We did not conduct any type of model selection but fitted a model based on expert knowledge of the system and the scope of our study, i.e. investigating how habitat selection was influenced by temperature. We obtained population level parameters by summarizing the individual coefficients of the iSSFs by running 500 bootstrap replications and calculating the mean as the population estimate. Likewise, 95% confidence intervals were calculated using the 2.5 and 97.5% quantile of the bootstrapped parameter distributions [92]. Each parameter value/selection coefficient shows the relative selection strength (RSS) [93] for a one unit increase of the corresponding covariate, given that all other covariates are equal. Further, to investigate seasonal patterns in habitat selection, we conducted a supporting analysis and refitted our models for three seasonal subsets (spring, summer and fall) based on data on the start and end of the vegetation period as well as the meteorological definition of the arrival of spring, summer and fall in our study area (see appendix for detailed definitions) [77, 94].

## Results

### Moose

**Fig. 3:**
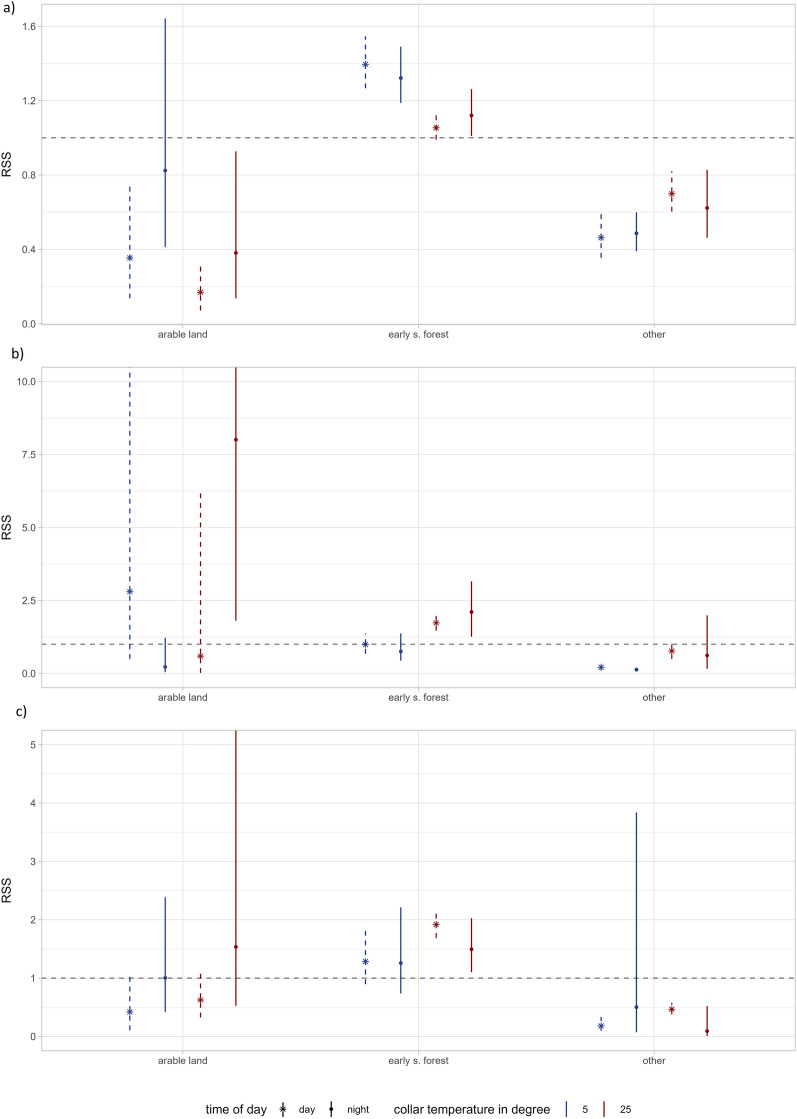
Relative selection strength (RSS) plots showing the interaction between temperature and land cover type habitat selection of a) moose (n = 27), b) red deer (n = 7) and c) roe deer (n = 12) between May–September during both day and night. Points show population level estimates obtained by bootstrapping the coefficients of individual models. Temperature is set to two constants 5 and 25 °C, represented by blue and red in order to make interpretation about selection between different temperatures easier. The y-axis represents the relative selection strength, which represents how much more likely or less likely an animal is to select for that habitat in comparison to forests (the reference category), indicated by the dashed line. The dashed line indicates no preference or avoidance. Error bars represent bootstrapped 95% confidence intervals around the population level estimates. Significant results are expressed when bars are not overlapping the dashed line. Time of day is indicated by a star and dashed line as day and dot and solid line as night

**Fig. 4:**
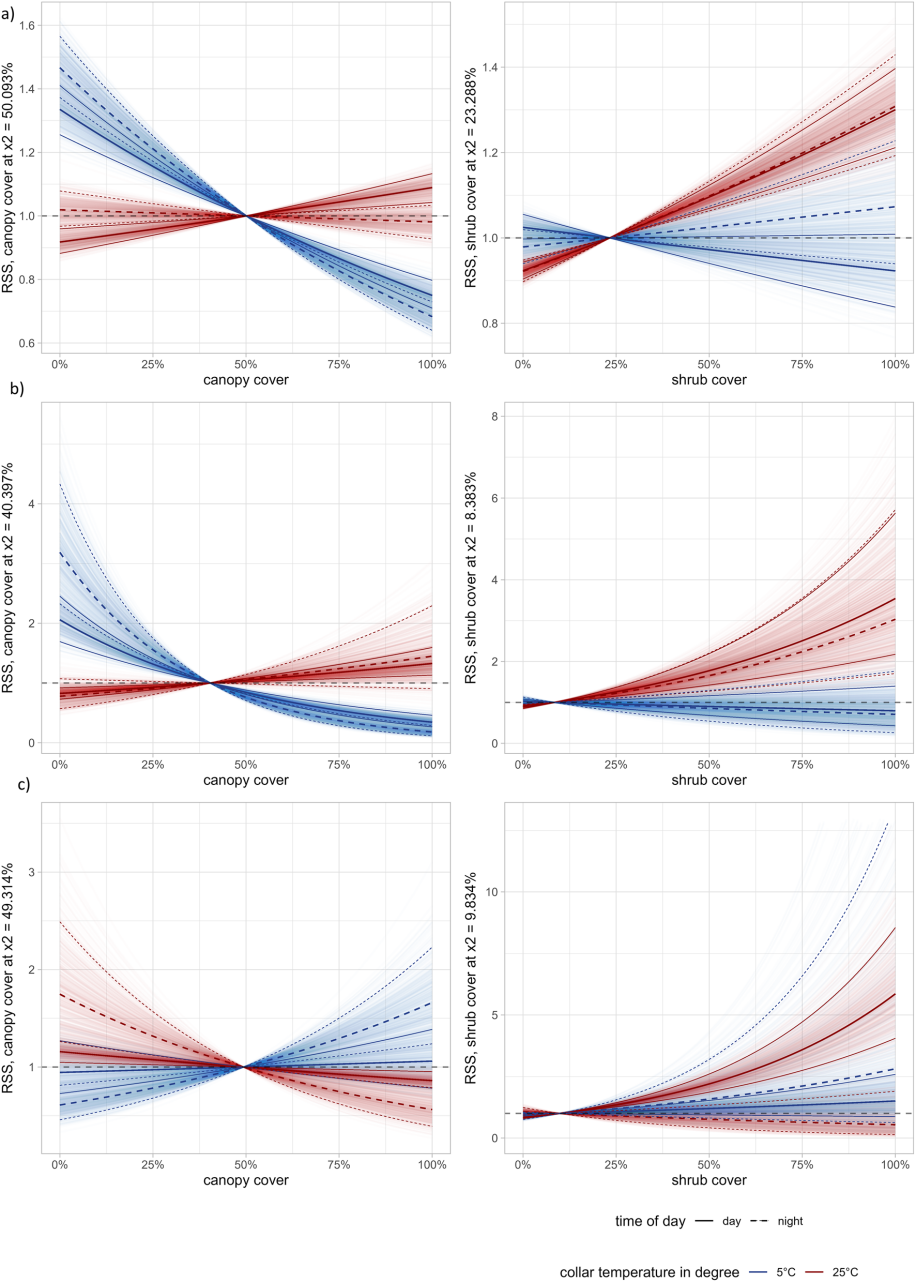
Relative selection strength (RSS) plots showing the interaction between temperature and canopy cover and shrub cover selection of a) moose (n = 27), b) red deer (n = 7) and c) roe deer (n = 12), between May–September during both day and night. Temperature is set to two constants 5 and 25 °C, represented by blue and red in order to make interpretation about selection between different temperatures easier. The y-axis represents the relative selection strength, which represents how much more likely or less likely an animal is to select for a habitat × 1 in relation to the average used canopy cover or shrub cover × 2. The dashed horizontal black line indicates no preference or avoidance. Error bands represent bootstrapped 95% confidence intervals around the population level estimates. Significant results are expressed when bands are not overlapping the dashed horizontal black line. Time of day is indicated by a solid and dashed line

Temperature influenced habitat selection of moose for canopy cover, shrub cover and all land cover types during day (Table 2). During night, temperature influenced selection canopy cover and shrub cover (Table 2). Moose avoided arable land compared to forests regardless of temperatures during day and night, although this was not significant regarding the coldest night-time temperatures (Fig. 3a). They selected for early successional forests compared to forests regardless of temperatures both during day and night (Fig. 3a). Moose avoided the habitat category “other”, regardless of temperature and time of day. During warmer days, moose selected for higher canopy cover. However, moose showed no selection for canopy cover during warmer nights and they even avoided higher canopy cover during colder nights and days (Fig. 4a). Moose selected for higher shrub cover during warmer days and nights but used it according to availability during colder days and nights (Fig. 4a). Moose showed differences in habitat selection between seasons (spring, summer and fall), however not always consistent between day and night (see appendix for supporting analysis). For example, they selected differently for canopy cover between seasons during daytime, however this result was not consistent during nights where they showed no seasonal influence on the selection (Fig. 1, appendix). Their selection of shrub cover, early successional forest and arable land showed seasonal differences that were consistent over day and night (Fig. 1, appendix).

**Table 2:** Coefficient estimates, standard errors (SE), relative selection strengths (RSS), and 95% confidence limits (lower, LCL; upper, UCL) on population level estimates of an iSSF of moose (n = 27) habitat selection during day and night in response to land cover types, canopy cover and shrub cover and their interactions with temperature at the start of the step (tc_start).

			Day					Night		
	Coefficient	SE	RSS	LCL	UCL	Coefficient	SE	RSS	LCL	UCL
Arable	**−1.521**	**0.080**	**0.219**	**0.095**	**0.410**	**−0.697**	**0.051**	**0.498**	**0.304**	**0.814**
Early s.forest	**0.150**	**0.005**	**1.162**	**1.108**	**1.221**	**0.171**	**0.007**	**1.186**	**1.110**	**1.277**
Other	**−0.501**	**0.013**	**0.606**	**0.532**	**0.689**	**−0.559**	**0.019**	**0.572**	**0.464**	**0.694**
Canopy cover	**−0.029**	**0.003**	**0.971**	**0.944**	**0.998**	**−0.096**	**0.003**	**0.909**	**0.879**	**0.937**
Shrub cover	**0.044**	**0.002**	**1.045**	**1.027**	**1.066**	**0.062**	**0.002**	**1.063**	**1.037**	**1.091**
Arable:tc_start	**−0.229**	**0.018**	**0.795**	**0.673**	**0.952**	−0.239	0.046	0.787	0.482	1.214
Early s.forest:tc_start	**−0.087**	**0.004**	**0.917**	**0.880**	**0.956**	−0.051	0.006	0.950	0.900	1.003
Other:tc_start	**0.128**	**0.009**	**1.137**	**1.035**	**1.247**	0.077	0.012	1.080	0.967	1.215
Canopy cover:tc_start	**0.077**	**0.002**	**1.080**	**1.061**	**1.097**	**0.074**	**0.002**	**1.077**	**1.053**	**1.102**
Shrub cover:tc_start	**0.033**	**0.001**	**1.033**	**1.020**	**1.049**	**0.019**	**0.002**	**1.019**	**1.001**	**1.038**

Confidence limits that overlap 1 (the reference level dividing selection and avoidance of habitats) imply that there was no clear avoidance or preference for this habitat. Significant avoidance or preferences are visualized by bold numbers. The data was z-score transformed

### Red deer

Temperature influenced red deer selection for canopy cover and shrub cover and for all land cover categories except arable land during the day (Table 3). During the night, temperature influenced all categories except shrub cover (Table 3).

**Table 3:** Coefficient estimates, standard errors (SE), relative selection strengths (RSS), and 95% confidence limits (lower, LCL; upper, UCL) on population level estimates of an iSSF of red deer (n = 7) habitat selection during day and night in response to land cover types, canopy cover and shrub cover and their interactions with temperature at the start of the step (tc_start).

			Day					Night		
	Coefficient	SE	RSS	LCL	UCL	Coefficient	SE	RSS	LCL	UCL
Arable	−0.089	0.343	0.915	0.415	3.708	**1.082**	**0.112**	**2.951**	**1.620**	**4.523**
Early s.forest	**0.393**	**0.022**	**1.482**	**1.340**	**1.680**	**0.457**	**0.054**	**1.580**	**1.161**	**1.981**
Other	**−0.622**	**0.095**	**0.537**	**0.313**	**0.842**	**−0.917**	**0.173**	**0.400**	**0.151**	**0.903**
Canopy cover	−0.058	0.020	0.943	0.851	1.052	−0.131	0.038	0.877	0.714	1.052
Shrub cover	**0.115**	**0.009**	**1.122**	**1.074**	**1.184**	**0.096**	**0.006**	**1.100**	**1.067**	**1.133**
Arable:tc_start	−0.413	0.297	0.662	0.130	1.662	**0.951**	**0.166**	**2.589**	**1.109**	**5.491**
Early s.forest:tc_start	**0.148**	**0.025**	**1.160**	**1.034**	**1.335**	**0.273**	**0.049**	**1.314**	**1.015**	**1.676**
Other:tc_start	**0.341**	**0.025**	**1.407**	**1.252**	**1.607**	**0.414**	**0.073**	**1.513**	**1.032**	**2.190**
Canopy cover:tc_start	**0.223**	**0.012**	**1.250**	**1.168**	**1.326**	**0.345**	**0.033**	**1.412**	**1.205**	**1.663**
Shrub cover:tc_start	**0.054**	**0.007**	**1.055**	**1.016**	**1.093**	0.053	0.013	1.054	0.991	1.129

Confidence limits that overlap 1 (the reference level dividing selection and avoidance of habitats) imply that there was no clear avoidance or preference for this habitat. Significant avoidance or preferences are visualized by bold numbers. The data was z-score transformed

Red deer used arable land to a similar degree as forest during daytime, however selected for arable land during warmer nights (Fig. 3b). Red deer selected early successional forests during warmer days and nights, while selecting for them similar as forests during colder temperatures (Fig. 3b).

Moreover, red deer selected for higher canopy cover during warmer temperatures during the day and night, however less strongly during the night (Fig. 4b). They avoided higher canopy cover during colder temperatures, both during day and night (Fig. 4b). Furthermore, red deer selected for higher shrub cover with increasing temperatures during day and night and used them according to average during colder temperatures (Fig. 4b). Season influenced red deer habitat selection for some land cover types however not always consistent between night and day (see appendix for detailed supporting analysis results). For arable land and early successional forest they showed seasonal differences in their selection, however, not consistent between day and night (Fig. 1, appendix). Their selection for canopy cover was influenced by season and was consistent over night and day (Fig. 1, appendix). Finally, they showed no influence of season on their selection of early successional forest (Fig. 1, appendix).

### Roe deer

Temperature influenced roe deer selection for shrub cover and the habitat class “other” during the day (Table 4). During the night, temperature influenced selection for canopy cover (Table 4).

**Table 4:** Coefficient estimates, standard errors (SE), relative selection strengths (RSS), and 95% confidence limits (lower, LCL; upper, UCL) on population level estimates of an iSSF of roe deer (n = 12) habitat selection during day and night in response to land cover types, canopy cover and shrub cover and their interactions with temperature at the start of the step (tc_start).

			Day					Night		
	Coefficient	SE	RSS	LCL	UCL	Coefficient	SE	RSS	LCL	UCL
Arable	−0.504	0.095	0.604	0.294	1.039	0.365	0.153	1.440	0.584	4.350
Early s.forest	**0.595**	**0.016**	**1.813**	**1.637**	**1.999**	**0.377**	**0.032**	**1.458**	**1.149**	**1.817**
Other	**−0.886**	**0.037**	**0.412**	**0.322**	**0.525**	**−2.182**	**0.291**	**0.113**	**0.011**	**0.442**
Canopy cover	−0.085	0.014	0.919	0.837	1.008	**−0.293**	**0.036**	**0.746**	**0.577**	**0.925**
Shrub cover	**0.225**	**0.009**	**1.253**	**1.182**	**1.343**	−0.054	0.024	0.947	0.799	1.103
Arable:tc_start	0.100	0.036	1.105	0.868	1.400	0.109	0.050	1.115	0.795	1.611
Early s.forest:tc_start	0.107	0.017	1.113	0.993	1.227	0.048	0.029	1.049	0.855	1.267
Other:tc_start	**0.253**	**0.020**	**1.288**	**1.104**	**1.471**	−0.500	0.175	0.607	0.172	1.547
Canopy cover:tc_start	−0.040	0.008	0.961	0.913	1.016	**−0.206**	**0.018**	**0.814**	**0.720**	**0.910**
Shrub cover:tc_start	**0.053**	**0.004**	**1.054**	**1.029**	**1.082**	−0.064	0.018	0.938	0.825	1.050

Confidence limits that overlap 1 (the reference level dividing selection and avoidance of habitats) imply that there was no clear avoidance or preference for this habitat. Significant avoidance or preferences are visualized by bold numbers. The data was z-score transformed

Roe deer avoided arable land regardless of temperatures both during day and night, however, the CI´s overlapped with the reference level during warmer nights (i.e. the selected for it to a similar degree as forests) (Fig. 3c). They selected for early successional forests compared to forests during warmer temperatures at day and night, but used them according to availability on colder nights and days (Fig. 3c). They avoided the habitat category “other”, during day and night, regardless of temperatures, however the CI´s overlapped with the reference level during cold night temperatures (Fig. 3c).

Roe deer showed no selection for canopy cover during colder days and nights (Fig. 4c), however, they avoided areas with high canopy cover during warmer temperatures during the day and night (Fig. 4c).

Roe deer strongly selected for higher shrub cover during warmer days, but showed no selection for shrub cover otherwise (Fig. 4c). Season influenced roe deer habitat selection but patterns were inconsistent between night and day (see appendix for detailed supporting analysis results). Their selection for canopy cover, early successional forest and shrub cover was influenced by season, however with different patterns between night and day (Fig. 1, appendix). Roe deer showed no difference in seasonal selection for arable land (Fig. 1, appendix).

## Discussion

### Species-specific responses to temperature

Our study revealed that temperature influenced the habitat selection for moose, red deer and roe deer coexisting in the same area, however with some differences between species. It suggests that temperature strongly influenced how these three deer species navigate and use the landscape. However, the species-specific differences imply that they respond differently to similar ambient temperatures and other mechanisms influencing habitat selection. Moose and red deer strongly selected for areas with more canopy cover during increasing temperatures during the day, however, opposite during the night. This goes in line with other studies, reporting on the selection of areas providing thermal shelter as temperatures increase [17, 18, 24, 95]. The fact that roe deer avoided areas with dense canopy cover during the night, and moose and red deer used areas with average canopy cover during night (i.e. not selecting for high canopy cover as strong as during warm days), also suggests patterns of thermoregulatory behavior, since dense forests trap heat [96, 97]. This suggests that they found better relief from the warmer night temperatures by selecting areas with lower canopy cover, hence, canopy cover may not be as important functioning as thermal refuge during the night. Additionally, this may also be reflected by the result that red deer selected for arable land compared to forests during warmer temperatures at night, and as such selected a more open habitat to cool down during warm night temperatures. Moreover, during colder temperatures, moose and red deer avoided areas with more canopy cover, but instead used more open areas where forage availability is likely higher [82, 98, 99].

### Thermal refuges and forage trade-offs

Moose and red deer selected areas with more shrub cover with increasing temperatures while no such pattern was found during colder days and nights.s. This could be due to several reasons. During summer, both moose and red deer spend a relatively large proportion of each 24 h-cycle bedded while ruminating and resting [53, 100, 101]. Furthermore, food intake is increased during spring and summer, and forage availability and quality are especially important for females since it is a crucial period with increased energy demands in order to produce milk for offspring and deposit fat in preparation for winter [54]. At the same time, they also need to respond to increasing temperatures by dissipating heat, and maintain thermal balance [102]. It is thus important for both moose and red deer to select areas where they can increase their heat loss both when bedded and when moving, while at the same time finding forage [103]. It has been found that thermal cover, proximity to browse and predator avoidance are all considered important drivers behind moose and red deer selection of beds [60, 102, 104–108]. This lends support to their strong selection of both areas with more canopy cover and shrub cover, providing forage and concealment from predators. Furthermore, it may be so that shrub cover below 3 and 2 m may serve as thermal shelter when both moose and red deer are lying down, something that would explain the divergent selection patterns of shrub cover between temperatures. [102] investigated bed site selection in moose and measured canopy cover just above the moose head when lying down (0.75 m). It may be so that our measurements of shrub cover can function as canopy cover for moose and red deer when bedded.

### Alternative roles of shrub cover−forage and concealment

Shrub cover may also function as an important concealment for hiding their offspring, something that is especially important during summer. Both moose and red deer calves are dependent on predator concealment in the beginning of their life [109, 111], especially red deer since they are a more distinct hider compared to moose, with their offspring being hidden directly after birth for approximately one to two weeks and then follows their mother closely [112, 114]. Habitats providing cover is important in the selection of bed sites for red deer offspring [107, 112, 114] as well as during the entire time when moose cows and red deer hinds are accompanied by their offspring [107, 115]. Moreover, deer species in this study area are also exposed to hunting during a part of the study period in autumn, something that has been shown to influence habitat selection in different deer species [19, 116, 117]. Hence, habitats providing concealment such as shrub cover, may also serve an important role during the hunting season. However, our results show that deer use denser shrub cover with increasing temperatures only. If hunting disturbance would be the driving factor behind the use of shrub cover, we should have seen strong selection also during colder temperatures.

### Seasonal influence on shrub cover selection

We found signs of moose selecting for areas with shrub cover and early successional forest during spring and summer, while using them to an average degree during fall. These patterns were also consistent between day and night (Table 1 appendix). This result suggests that it may be so that selection for areas with more shrub cover, such as younger forests, can be due to phenological changes in forage quality, as protein concentration and digestibility in leaves generally decrease from spring to autumn in this region [118]. However, it may also be because of other driving mechanisms changing between summer and fall, such as hunting pressure, as discussed above.

### Selection for early successional forests

Moose selected for early successional forests compared to forests regardless of temperature both during day and night. Red deer also preferred early successional forests, however not significantly during colder temperatures. Hence, these areas seem to be preferred habitats in comparison with more mature forests. This is most likely because early successional forests and clear cuts host large amounts of forage [119, 121]. Furthermore, planted clear cuts may also provide shrub cover and lower canopy cover, as well as local canopy cover by a few mature trees left after harvest. Hence it is important to remember that a clear cut is in this study defined as any forest land with vegetation below 5 m. These habitats may provide local thermal shelter for red deer and moose, which can explain why they are used also at warmer temperatures, and especially preferred by red deer. Furthermore, this goes in line with the shrub cover results, where moose and red deer used areas with more shrub cover during increasing temperatures, which could potentially be denser early successional forests or clear cuts.

### Different selection patterns by roe deer compared to moose and red deer

Roe deer showed divergent patterns of selection for canopy cover compared to the other species, as warmer temperature did not influence the roe deer’ selection of canopy cover during the day or night. This could potentially be because roe deer, being relatively small-bodied, may be less heat sensitive than moose and roe deer and are therefore not in the need for thermal refuge to a similar degree during the same temperatures.

However, similar to moose and red deer, roe deer selected areas with more shrub cover during increasing temperatures, however only during the day. It may be so that shrub cover (below 2 m) is sufficient for acting as thermal shelter for this smaller species during warmer daily temperatures, especially so when roe deer are bedded. It has been shown that roe deer prefer to bed down below dense canopy cover, measured with lower heights than our canopy cover measurement [122]. Moreover, shrub cover may also be especially important for roe deer females during this period, serving as hiding cover and predator concealment for their fawns. Roe deer fawns are especially vulnerable to fox predation [123], and females hide their fawns for as long as four weeks while the mother is foraging and coming back to provide milk multiple times during the day [113, 123]. Bed sites where fawns are placed are chosen to decrease the predation risk, i.e. in areas with a high understory cover [123, 125] and studies suggest that roe deer fawns in open habitats are more vulnerable to predation, as visibility increases [123, 126, 127]. Bongi et al., [128] found that during summer, roe deer used habitats where visibility was lower, most likely because females seldom move far away from their hidden fawns [129]. This could further explain roe deer’s strong selection for habitats with more shrub cover. However, the importance of shrub cover as predator concealment for fawns would most likely be seasonally driven, i.e. being most important right after calving in spring. When breaking down our results to finer seasonal grain in the supporting analysis (see appendix), we found that roe deer showed no seasonal influence on selection of shrub cover during daytime, while selecting for more shrub cover during spring nights (Table 5, appendix). This result implies that their selection of shrub cover variation was most likely influenced by temperature on finer scales, even though seasonal changes in forage availability or the need for predator concealment also may have played a role.

Similarly, we found no seasonal patterns in habitat selection of early successional forests for roe deer that were consistent between day and night, implying that the influence of temperature on early successional forest selection was not primarily driven by seasonal changes in phenology and forage availability, but instead finer scale temperature changes (Fig. 1 appendix). Generally, roe deer selected for early successional forests during warm temperatures, both during day and night. This is most likely because these habitats provide high availability of food, but also can provide both concealment for offspring as well as some thermal shelter for smaller roe deer.

### Future directions and study limitations

Future studies would ideally include information about additional important variables influencing habitat section in these species in order to get a better understanding about the driving factors. Calving dates and difference between individuals accompanied by calves and those without would make it possible to better determine if habitat selection was indeed mainly driven by temperature, or also influenced by calving. Furthermore, information about movement patterns and speed would give ideas of whether the positions of animals were used as resting sites. Additionally, to gain a better understanding of species interactions and competition, density dependent analyses would be valuable. Finally, the sample size of red deer would also be larger. We had GPS data of only seven individual red deer, which likely leads to variation and uncertainty within our study. However, the results align with the literature on the behaviour of this species [130]. Moreover, the lack of GPS positions in waterbodies made it impossible for us to separately analyse the use of potentially important thermal refuges, such as wetlands. This would however be interesting and important to investigate in future studies since it has been suggested that wet substrates function as important thermal refuges where moose can cool off [55, 102, 131], something that has also been found in roe deer [122]. Next, there might be confounding variation in collar temperature due to expended heat during the movement process (i.e., increased collar temperature due to higher energy expenditure when animals move more), which we did not consider within the framework of this study. Furthermore, more detailed fine scale information on forage availability and forage quality of the GPS positions of the animals over the study period would give valuable information on the mechanisms behind habitat selection and whether it is driven by phenological changes in forage availability or quality on smaller scales. This would especially be the case when trying to tease apart the selection of habitats that simultaneously provide forage, thermal refuge and predator concealment.

## Conclusions

Our results provide insights into the driving mechanisms behind habitat selection and may also have important implications for management. In general, our findings suggest that management actions that influence the cover of different vegetation strata directly influence ungulates’ ability to respond to increasing temperatures, as well as several other stimuli and fulfilling internal needs. However, future research is needed to confirm these relationships in a broader context and to get a better understanding of the driving mechanisms behind habitat selection. Nonetheless, management strategies that aim to favour forage availability while maintaining local shading may provide win–win situations for ungulates. This could be done, for example by promoting habitats like mature open forests that provide local shading opportunities from large trees, while also allowing for partial light penetration which is important for a flourishing field layer containing important food items for all three ungulate species [59, 132]. 

## Supplementary Information

Additional file 1.

## Data Availability

Data and code have been archived in the Open Science Framework (OSF) repository and are available via: 10.17605/OSF.IO/7NXGD.
