# Supplementary material for: Temperature mediated habitat selection in sympatric deer species with varying body size: thermal cover and forage availability as potential drivers
Source: Mov Ecol. 2025 Jul 22;13:52. doi: 10.1186/s40462-025-00581-2 (PMC12281807; doi:10.1186/s40462-025-00581-2)
Supplement: Supplementary file 1 — Additional file 1. [file 40462_2025_581_MOESM1_ESM.docx]

**Appendix:**

Supporting analysis conducted on seasonal habitat selection.

1. **Method**

To investigate seasonal patterns in habitat selection we analysed habitat selection using integrated Step Selection Functions (iSSF) (Avgar *et al.* 2016). We did this to analyse whether selection for land cover types, shrub cover and canopy cover is dependent on season. We homogenized sampling rates for all species by resampling to a three-hour sampling rate. We fitted iSSFs for every individual using the amt-package (Signer *et al*., 2019), generating 50 random available steps (Thurfjell *et al*., 2014) for every observed step. Available steps were generated using a gamma distribution to fit step length and a Von Mises distribution for turning angles. We extracted environmental covariates at the end of each step. We used canopy and shrub cover as continuous variables in the model in addition to the land cover factor (with the four categories: arable, early successional forest, forest and other). We used forest as the reference habitat. We performed z-score transformation on canopy cover and shrub cover (Zuur *et al.*, 2010). We further included the step length, the logarithm of the step length and the cosine of the turning angle as covariates in the model to account for the underlying movement process of iSSFs (Avgar *et al.*, 2016). The data was split into three seasonal subsets (spring, summer and fall). The seasonal division was based on the start and end of the vegetation period in our study area and the meteorological entrance of spring, summer and fall, where start of the vegetation period is defined as when the daily average temperature exceeds 5 degrees celsius for six consecutive days and the end of the vegetation period is defined as the day before the first six day period after July 1^st^, during which all six days have a daily average temperature below 5 degrees celsius. The meteorological definition of the entrance of spring, summer and fall is defined as when the daily average temperature exceeds a certain threshold for 5 executive days, spring=above 0 degrees celsius, summer = above 10 degrees celsius and fall = below 10 degrees celsius (SMHI 2024a & 2024b). Thus, spring was defined from the 1st of May to the 31st of May, the summer 1st of June to the 14th of September and fall from 15th of September to the 30th of September. As some animals had not enough observed steps to ensure reliable coefficient estimation (i.e., high coefficients associated with high standard errors), we instead summarized coefficients using inverse weighted variance (IWV) - regression (see Graf *et al.,* 2024; Picardi *et al*., 2022), as it gives lower weights to coefficients with higher uncertainty. We had not enough data to reliably estimate coefficients for habitat selection of red deer in the fall season.

selection we analysed habitat selection using integrated Step Selection Functions (iSSF) (Avgar *et al.* 2016).

**Results**

**Moose**

Moose avoided arable land compared to forest during both day and night in spring and summer and showed no selection compared to forest during fall daytime (table 1 & 2, fig 1). However, during the night in fall, they instead selected for it compared to forests (table 2, fig 1). They selected for early successional forest during all seasons both day and night, however not significantly so during fall (fig 1). They showed no seasonal difference in their selection of the land cover category “other”, regardless of time of day (fig 1). Moose avoided areas with high canopy cover during daytime periods, regardless of season, however not significantly so during spring (table 1, fig 1). During the night, they avoided areas with more canopy cover during all seasons (table 2). Finally, moose selected for areas with more shrub cover during spring and summer both during day and night and using it according to average during fall (fig 1).

*Table 1. Coefficient estimates, standard errors (SE), relative selection strength (RSS), and 95% confidence limits (lower, LCL; upper, UCL) on population level estimates of an iSSF of moose habitat selection during three different seasons during the day, in response to land cover types, canopy cover and shrub cover. Confidence limits that overlap 1 (the reference level dividing selection and avoidance of habitats) imply that there was no clear avoidance or preference for this habitat. Clear avoidance or preference is indicated with bold numbers. Season is divided into spring (1^st^ of May – 31^st^ of May), summer (1^st^ of June – 15^th^ of September) and fall (16^th^ of September – 30^th^ of September). Early s. forest stands for early successional forest.*

|  |  |  | Spring |  |  |  |  | Summer |  |  |  | Fall |  |  |  |
| --- | --- | --- | --- | --- | --- | --- | --- | --- | --- | --- | --- | --- | --- | --- | --- |
|  | Coefficient | SE | RSS | LCL | UCL | Coefficient | SE | RSS | LCL | UCL | Coefficient | SE | RSS | LCL | UCL |
| Arable | **-0.871** | **0.129** | **0.418** | **0.325** | **0.539** | **-1.008** | **0.120** | **0.364** | **0.288** | **0.426** | 0.459 | 0.195 | 1.576 | 1.074 | 2.310 |
| Early s. forest | **0.165** | **0.029** | **1.179** | **1.113** | **1.249** | **0.140** | **0.024** | **1.150** | **1.096** | **1.207** | 0.111 | 0.072 | 1.117 | 0.969 | 1.288 |
| Other | **-0.396** | **0.089** | **0.672** | **0.564** | **0.800** | **-0.160** | **0.067** | **0.851** | **0.745** | **0.972** | **-0.354** | **0.139** | **0.701** | **0.536** | **0.917** |
| Canopy cover | -0.002 | 0.015 | 0.997 | 0.967 | 1.028 | **-0.045** | **0.016** | **0.955** | **0.923** | **0.987** | **-0.130** | **0.058** | **0.877** | **0.782** | **0.984** |
| Shrub cover | **0.045** | **0.009** | **1.046** | **1.027** | **1.064** | **0.076** | **0.016** | **1.078** | **1.045** | **1.113** | 0.021 | 0.033 | 1.021 | 0.955 | 1.091 |

*Table 2. Coefficient estimates, standard errors (SE), relative selection strengths (RSS), and 95% confidence limits (lower, LCL; upper, UCL) on population level estimates of an iSSF of moose habitat selection during three different seasons during the night, in response to land cover types, canopy cover and shrub cover. Confidence limits that overlap 1 (the reference level dividing selection and avoidance of habitats) imply that there was no clear avoidance or preference for this habitat. Clear avoidance or preference is indicated with bold numbers. Season is divided into spring (1^st^ of May – 31^st^ of May), summer (1^st^ of June – 15^th^ of September) and fall (16^th^ of September – 30^th^ of September). Early s. forest stands for early successional forest.*

|  |  |  | Spring |  |  |  |  | Summer |  |  |  | Fall |  |  |  |
| --- | --- | --- | --- | --- | --- | --- | --- | --- | --- | --- | --- | --- | --- | --- | --- |
|  | Coefficient | SE | RSS | LCL | UCL | Coefficient | SE | RSS | LCL | UCL | Coefficient | SE | RSS | LCL | UCL |
| Arable | **-0.550** | **0.131** | **0.576** | **0.445** | **0.746** | **-0.545** | **0.122** | **0.579** | **0.455** | **0.737** | **0.694** | **0.215** | **2.001** | **1.311** | **3.055** |
| Early s. forest | **0.228** | **0.039** | **1.257** | **1.164** | **1.357** | **0.210** | **0.047** | **1.234** | **1.123** | **1.355** | 0.096 | 0.152 | 1.101 | 0.816 | 1.486 |
| Other | **-0.431** | **0.086** | **0.649** | **0.547** | **0.769** | -0.128 | 0.090 | 0.879 | 0.737 | 1.048 | **-0.595** | **0.201** | **0.551** | **0.371** | **0.818** |
| Canopy cover | **-0.064** | **0.019** | **0.937** | **0.901** | **0.974** | **-0.119** | **0.021** | **0.887** | **0.850** | **0.926** | **-0.217** | **0.073** | **0.804** | **0.696** | **0.928** |
| Shrub cover | **0.053** | **0.012** | **1.055** | **1.028** | **1.082** | **0.036** | **0.016** | **1.037** | **1.003** | **1.072** | -0.013 | 0.047 | 0.986 | 0.899 | 1.082 |

**Red deer**

In daytime, red deer selected for arable land compared to forest only during both spring and summer, however only significantly so during summer (table 3, fig 1). During the night, they selected for it to a similar degree as forest both during spring and summer (table 3, table 4). They preferred early successional forest compared to more mature forest regardless of season during the day, however, during the night they only selected for it during the summer and showed no significant preference nor avoidance during spring (fig 1). Red deer avoided areas with more canopy cover during spring days, while selecting for them as average during summer days (table 3, fig1). During night they also avoided areas with high canopy cover during spring, while selecting for them as average during summer (table 4). Furthermore, they avoided the land cover category “other” regardless of season, however, overlapping with the reference line (indicating no preference nor avoidance) during summer daytime and nighttime. Red deer showed no seasonal influence on the selection of shrub cover, where they selected for areas with more shrub cover regardless of season and time of day. (fig 1). Note that the observations for red deer in the fall period were too few in order to reach a converging model.

Table 3. Coefficient estimates, standard errors (SE), relative selection strengths (RSS), and 95% confidence limits (lower, LCL; upper, UCL) on population level estimates of an iSSF of red deer habitat selection during three different seasons during the day, in response to land cover types, canopy cover and shrub cover. Confidence limits that overlap 1 (the reference level dividing selection and avoidance of habitats) imply that there was no clear avoidance or preference for this habitat. Clear avoidance or preference is indicated with bold numbers. Note that there was not sufficient data for a converging model during the fall period for red deer. Season is divided into spring (1^st^ of May – 31^st^ of May), summer (1^st^ of June – 15^th^ of September) and fall (16^th^ of September – 30^th^ of September). Early s. forest stands for early successional forest.

|  |  |  | Spring |  |  |  |  | Summer |  |  |  | Fall |  |  |  |
| --- | --- | --- | --- | --- | --- | --- | --- | --- | --- | --- | --- | --- | --- | --- | --- |
|  | Coefficient | SE | RSS | LCL | UCL | Coefficient | SE | RSS | LCL | UCL | Coefficient | SE | RSS | LCL | UCL |
| Arable | 0.563 | 0.306 | 1.756 | 0.963 | 3.199 | **0.980** | **0.153** | **2.665** | **1.971** | **3.603** | - | - | - | - | - |
| Early s. forest | **0.339** | **0.073** | **1.403** | **1.216** | **1.620** | **0.491** | **0.108** | **1.635** | **1.321** | **2.022** | - | - | - | - | - |
| Other | **-1.004** | **0.230** | **0.366** | **0.233** | **0.575** | -0.348 | 0.291 | 0.705 | 0.398 | 1.248 | - | - | - | - | - |
| Canopy cover | **-0.179** | **0.043** | **0.836** | **0.767** | **0.911** | 0.067 | 0.082 | 1.069 | 0.909 | 1.258 | - | - | - | - | - |
| Shrub cover | **0.053** | **0.011** | **1.055** | **1.032** | **1.078** | **0.143** | **0.047** | **1.154** | **1.052** | **1.266** | - | - | - | - | - |

*Table 4. Coefficient estimates, standard errors (SE), relative selection strengths (RSS), and 95% confidence limits (lower, LCL; upper, UCL) on population level estimates of an iSSF of red deer habitat selection during three different seasons during the night, in response to land cover types, canopy cover and shrub cover. Confidence limits that overlap 1 (the reference level dividing selection and avoidance of habitats) imply that there was no clear avoidance or preference for this habitat. Clear avoidance or preference is indicated with bold numbers. Note that there was not sufficient data for a converging model during the fall period for red deer. Season is divided into spring (1^st^ of May – 31^st^ of May), summer (1^st^ of June – 15^th^ of September) and fall (16^th^ of September – 30^th^ of September). Early s. forest stands for early successional forest.*

|  |  |  | Spring |  |  |  |  | Summer |  |  |  | Fall |  |  |  |
| --- | --- | --- | --- | --- | --- | --- | --- | --- | --- | --- | --- | --- | --- | --- | --- |
|  | Coefficient | SE | RSS | LCL | UCL | Coefficient | SE | RSS | LCL | UCL | Coefficient | SE | RSS | LCL | UCL |
| Arable | -0.084 | 0.407 | 0.919 | 0.413 | 2.045 | 0.664 | 0.475 | 1.942 | 0.764 | 4.933 | - | - | - | - | - |
| Early s. forest | -0.054 | 0.170 | 0.946 | 0.677 | 1.322 | **0.471** | **0.236** | **1.602** | **1.008** | **2.546** | - | - | - | - | - |
| Other | **-1.587** | **0.236** | **0.204** | **0.128** | **0.324** | -0.592 | 0.517 | 0.552 | 0.200 | 1.524 | - | - | - | - | - |
| Canopy cover | **-0.645** | **0.089** | **0.524** | **0.440** | **0.625** | -0.053 | 0.113 | 0.947 | 0.758 | 1.184 | - | - | - | - | - |
| Shrub cover | **0.095** | **0.033** | **1.100** | **1.030** | **1.174** | **0.089** | **0.024** | **1.093** | **1.042** | **1.146** | - | - | - | - | - |

**Roe deer**

Roe deer showed no seasonal influence on selection of arable land during day nor night; hence they selected for it similar to forests during all periods (fig 1). They selected for early successional forests regardless of season during the day and night, however, overlapping with the reference level during spring and fall nighttime (i.e. showing no preference nor avoidance during these periods) (fig 1). Roe deer avoided areas with more canopy cover during summer days and nights. During fall, they instead selected for these areas, however not significantly so during night time (fig 1). Their selection for shrub cover showed no seasonal influence during daytime periods where they selected for areas with more shrub cover in all seasons (table 5). During nighttime, they selected for it during spring, and used it as average during summer and fall (table 6).

*Table 5. Coefficient estimates, standard errors (SE), relative selection strengths (RSS), and 95% confidence limits (lower, LCL; upper, UCL) on population level estimates of an iSSF of roe deer habitat selection during three different seasons during the day, in response to land cover types, canopy cover and shrub cover. Confidence limits that overlap 1 (the reference level dividing selection and avoidance of habitats) imply that there was no clear avoidance or preference for this habitat. Clear avoidance or preference is indicated with bold numbers. Season is divided into spring (1^st^ of May – 31^st^ of May), summer (1^st^ of June – 15^th^ of September) and fall (16^th^ of September – 30^th^ of September). Early s. forest stands for early successional forest.*

|  |  |  | Spring |  |  |  |  | Summer |  |  |  | Fall |  |  |  |
| --- | --- | --- | --- | --- | --- | --- | --- | --- | --- | --- | --- | --- | --- | --- | --- |
|  | Coefficient | SE | RSS | LCL | UCL | Coefficient | SE | RSS | LCL | UCL | Coefficient | SE | RSS | LCL | UCL |
| Arable | 0.136 | 0.125 | 1.145 | 0.895 | 1.466 | 0.034 | 0.129 | 1.034 | 0.802 | 1.334 | 0.035 | 0.427 | 1.036 | 0.447 | 2.397 |
| Early s. forest | **0.450** | **0.082** | **1.568** | **1.335** | **1.841** | **0.698** | **0.059** | **2.011** | **1.790** | **2.259** | **0.675** | **0.179** | **1.965** | **1.382** | **2.794** |
| Other | **-0.842** | **0.140** | **0.430** | **0.326** | **0.566** | **-0.542** | **0.111** | **0.581** | **0.467** | **0.722** | -0.579 | 0.303 | 0.560 | 0.309 | 1.015 |
| Canopy cover | -0.078 | 0.066 | 0.924 | 0.811 | 1.054 | **-0.106** | **0.406** | **0.899** | **0.830** | **0.973** | **0.366** | **0.156** | **1.443** | **1.061** | **1.962** |
| Shrub cover | **0.247** | **0.032** | **1.281** | **1.203** | **1.364** | **0.255** | **0.032** | **1.290** | **1.211** | **1.375** | **0.117** | **0.049** | **1.125** | **1.022** | **1.238** |

*Table 6. Coefficient estimates, standard errors (SE), relative selection strengths (RSS), and 95% confidence limits (lower, LCL; upper, UCL) on population level estimates of an iSSF of roe deer habitat selection during three different seasons during the night, in response to land cover types, canopy cover and shrub cover. Confidence limits that overlap 1 (the reference level dividing selection and avoidance of habitats) imply that there was no clear avoidance or preference for this habitat. Season is divided into spring (1^st^ of May – 31^st^ of May), summer (1^st^ of June – 15^th^ of September) and fall (16^th^ of September – 30^th^ of September). Early s. forest stands for early successional forest.*

|  |  |  | Spring |  |  |  |  | Summer |  |  |  | Fall |  |  |  |
| --- | --- | --- | --- | --- | --- | --- | --- | --- | --- | --- | --- | --- | --- | --- | --- |
|  | Coefficient | SE | RSS | LCL | UCL | Coefficient | SE | RSS | LCL | UCL | Coefficient | SE | RSS | LCL | UCL |
| Arable | 0.517 | 0.411 | 1.163 | 0.519 | 2.607 | 0.368 | 0.258 | 1.445 | 0.871 | 2.397 | 0.191 | 0.400 | 1.211 | 0.552 | 2.654 |
| Early s. forest | 0.178 | 0.230 | 1.195 | 0.760 | 1.879 | **0.383** | **0.120** | **1.468** | **1.160** | **1.857** | 0.308 | 0.222 | 1.361 | 0.879 | 2.106 |
| Other | -0.170 | 0.284 | 0.842 | 0.482 | 1.471 | **-0.771** | **0.159** | **0.462** | **0.338** | **0.631** | 0.093 | 0.265 | 1.098 | 0.653 | 1.847 |
| Canopy cover | -0.205 | 0.113 | 0.814 | 0.652 | 1.017 | **-0.235** | **0.081** | **0.790** | **0.673** | **0.926** | 0.251 | 0.141 | 1.285 | 0.974 | 1.694 |
| Shrub cover | **0.206** | **0.057** | **1.229** | **1.098** | **1.376** | 0.033 | 0.054 | 1.033 | 0.929 | 1.149 | 0.130 | 0.095 | 1.138 | 0.944 | 1.373 |


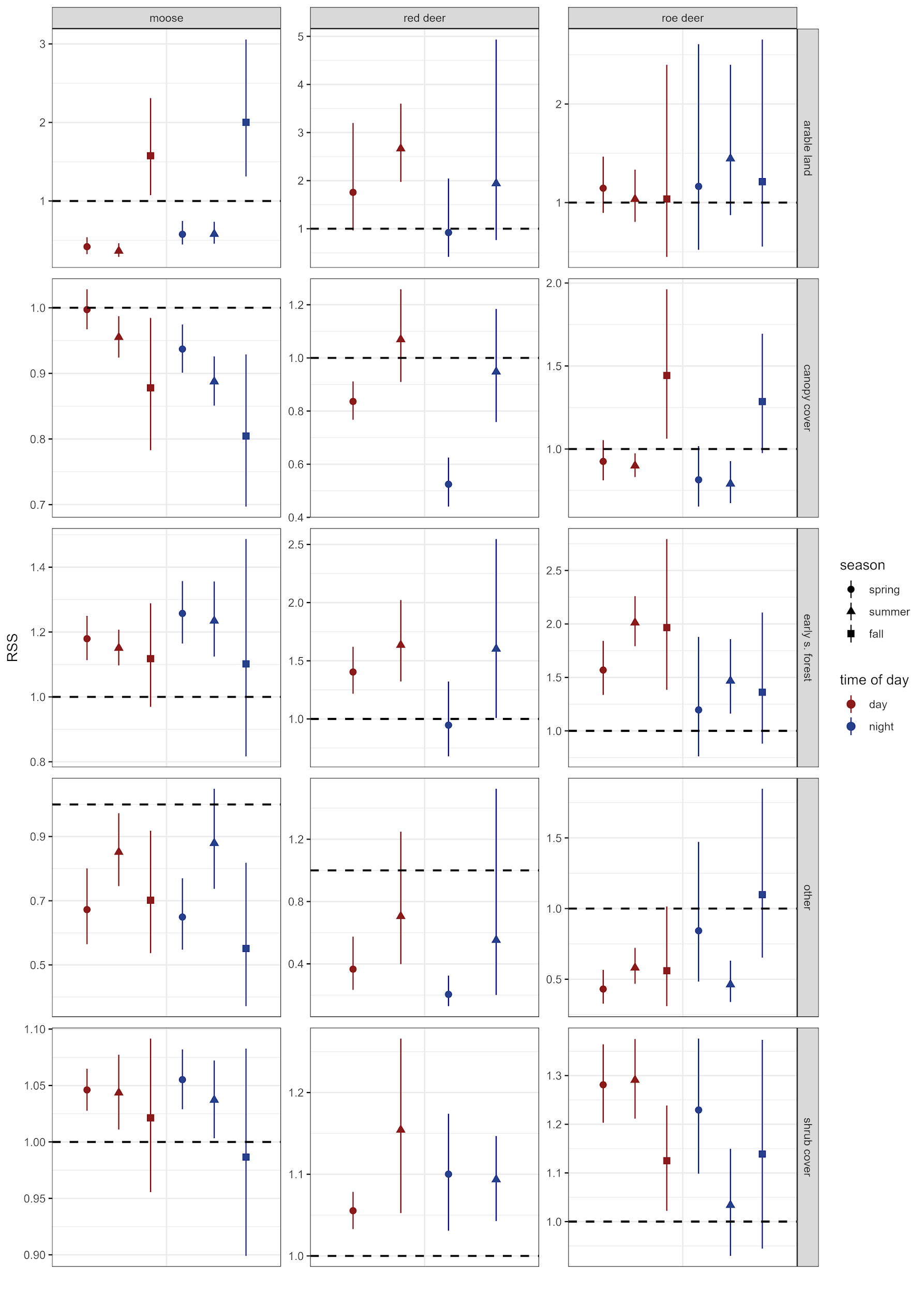

Figure 1. Relative selection strength (RSS) plots showing the influence of season on habitat selection of moose, red deer and roe deer, divided into day and night. Season is divided into spring (1^st^ of May – 31^st^ of May), summer (1^st^ of June – 15^th^ of September) and fall (16^th^ of September – 30^th^ of September). This division is based on the start and end of the vegetation period in our study area, as well as the entrance of the meteorological spring, summer and fall in our study area. Points show population level estimates obtained by IVW-Regression. The y-axis represents the relative selection strength, which represents how much more likely or less likely an animal is to select for that habitat in comparison to forests for the plots looking at arable land, other and clear cut, as well as for the average canopy cover and shrub cover, indicated by the dashed line. The dashed line indicates no preference or avoidance. Error bars represent 95% confidence intervals around the population level estimates. Significant results are expressed when bars are not overlapping the dashed line. Time of day is indicated by red or blue color, while season is indicated by circle, triangle or square. Early s. forest stands for early successional forest. Note that we had not enough data to reliably estimate coefficients for habitat selection of red deer in the fall season.

*Table 7. Original NMD code and classification of the different habitat classes used to create new classes that was used in the final models. Early s. forest stands for early successional forest.*

| **NMD code** | **NMD class** | **New classes** |
| --- | --- | --- |
| 2 | Open wetland | Other |
| 3 | Arable land | Arable |
| 41 | Non-vegetated other open land | Early s. forest |
| 42 | Vegetated other open land | Early s. forest |
| 51 | Artificial surfaces, buildings | Other |
| 52 | Artificial surfaces, not building or road/rail-way | Other |
| 53 | Artificial surfaces, road/rail-way | Other |
| 61 | Inland water | Other |
| 62 | Marine water | Other |
| 111 | Pine forest not on wetland | Forest |
| 112 | Spruce forest not on wetland | Forest |
| 113 | Mixed coniferous not on wetland | Forest |
| 114 | Mixed forest not on wetland | Forest |
| 115 | Deciduous forest not on wetland | Forest |
| 116 | Deciduous hardwood forest not on wetland | Forest |
| 117 | Deciduous forest with deciduous hardwood forest not on wetland | Forest |
| 118 | Temporarily non-forest not on wetland | Early s. forest |
| 121 | Pine forest on wetland | Forest |
| 122 | Spruce forest on wetland | Forest |
| 123 | Mixed coniferous on wetland | Forest |
| 124 | Mixed forest on wetland | Forest |
| 125 | Deciduous forest on wetland | Forest |
| 126 | Deciduous hard-wood forest on wetland | Forest |
| 127 | Deciduous forest with deciduous hardwood forest on wetland | Forest |
| 128 | Temporarily non-forest on wetland | Early s. forest |

*Table 8. Correlation matrix between the two variables shrub cover and canopy cover for each land cover type used in the anlaysis. Multicollinerarity is tested with used and available steps of each species separately in every habitat using spearman rank correlation.*

|  |  |  | **Canopy cover** | | | |
| --- | --- | --- | --- | --- | --- | --- |
| Moose | Forest | **Shrub cover** | 0.21 |  |  |  |
|  | Arable land |  |  | 0.51 |  |  |
|  | Early successional forest |  |  |  | 0.52 |  |
|  | Other |  |  |  |  | 0.60 |
| Red deer | Forest | **Shrub cover** | 0.19 |  |  |  |
|  | Arable land |  |  | 0.50 |  |  |
|  | Early successional forest |  |  |  | 0.46 |  |
|  | Other |  |  |  |  | 0.62 |
| Roe deer | Forest | **Shrub cover** | 0.22 |  |  |  |
|  | Arable land |  |  | 0.45 |  |  |
|  | Early successional forest |  |  |  | 0.35 |  |
|  | Other |  |  |  |  | 0.66 |

*Table 9. Correlations between Canopy cover and collar temperature or shrub cover. Columns Moose, Roe deer and Red deer show the respective pearson correlation coefficient r.*

| *Habitat class* | *Time of day* |  |  | *Moose* | *Roe deer* | *Red deer* |
| --- | --- | --- | --- | --- | --- | --- |
| forest | day |  |  | 0.100 | -0.013 | 0.130 |
| arable |  |  |  | 0.042 | 0.019 | -0.059 |
| clear_cut |  |  |  | 0.150 | -0.010 | 0.250 |
| other |  |  | Collar | 0.100 | 0.140 | 0.230 |
| forest | night |  | temperature | 0.150 | -0.014 | 0.300 |
| arable |  |  |  | 0.068 | -0.037 | 0.064 |
| clear_cut |  |  |  | 0.160 | -0.040 | 0.240 |
| other |  | Canopy |  | 0.056 | 0.140 | 0.250 |
| forest | day | cover |  | 0.240 | 0.065 | 0.130 |
| arable |  |  |  | 0.430 | 0.350 | 0.480 |
| clear_cut |  |  |  | 0.390 | 0.049 | 0.260 |
| other |  |  | Shrub | 0.520 | 0.250 | 0.120 |
| forest | night |  | cover | 0.230 | 0.089 | 0.200 |
| arable |  |  |  | 0.420 | 0.250 | 0.081 |
| clear_cut |  |  |  | 0.420 | 0.036 | 0.310 |
| other |  |  |  | 0.520 | 0.010 | 0.040 |


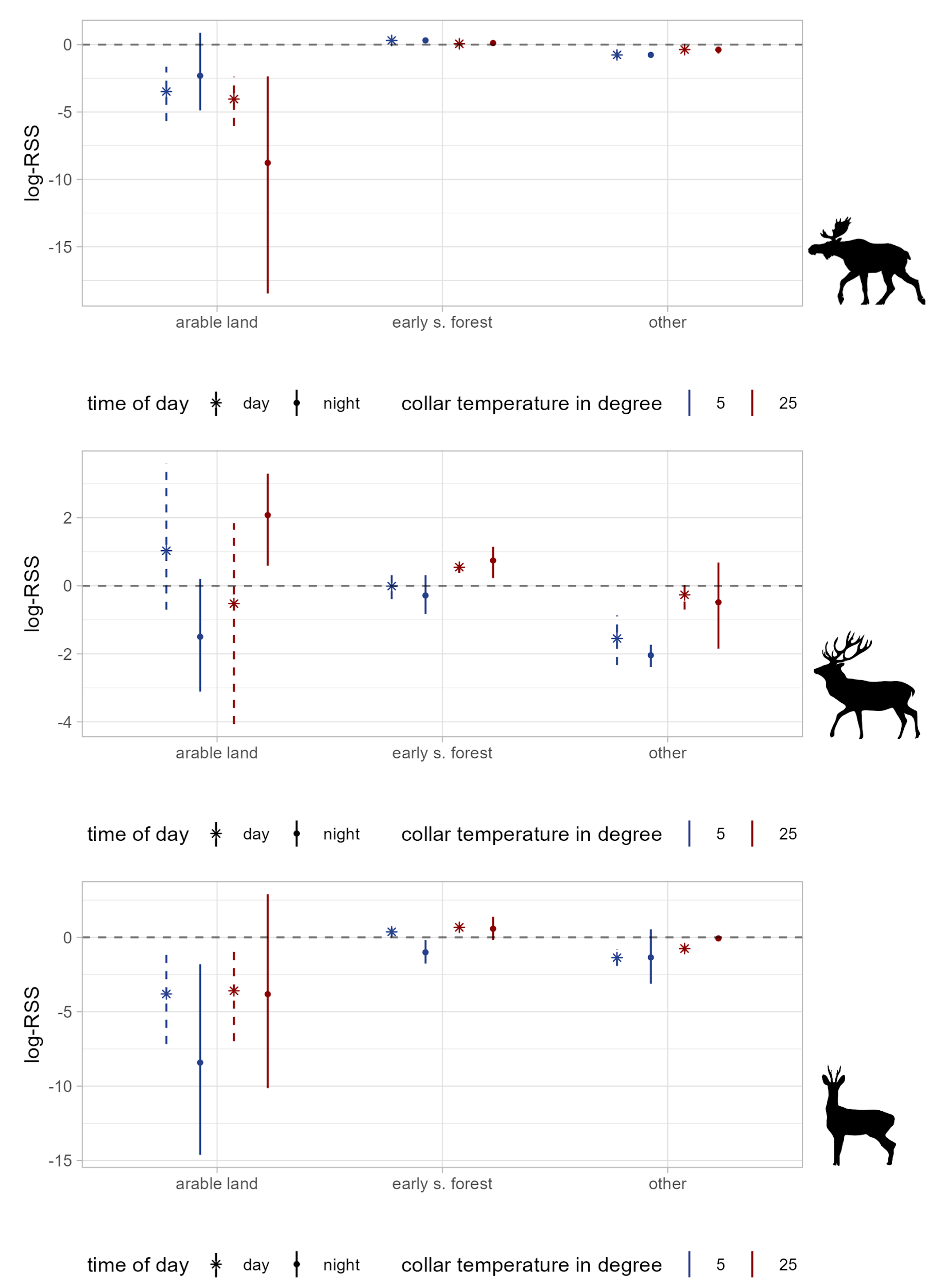


Figure 3. Relative selection strength on the log-scale (log-RSS) plots showing the interaction between temperature habitat classes of a) moose (n=27), b) red deer (n=7) and c) roe deer (n=12), between May-September during both day and night. Temperature is set to two constants 5 and 25 °C, represented by blue and red in order to make interpretation about selection between different temperatures easier. The y-axis represents the relative selection strength (on the log-scale for visual interpretation), which represents how much more likely or less likely an animal is to select for that habitat in comparison to the average canopy cover or shrub cover. The dashed horizontal black line indicates no preference or avoidance. Error bands represent bootstrapped 95% confidence intervals around the population level estimates. Significant results are expressed when bands are not overlapping the dashed horizontal black line. Time of day is indicated by a solid and dashed line.
